# Hydrogel microrobots for biomedical applications

**DOI:** 10.3389/fchem.2024.1416314

**Published:** 2024-05-22

**Authors:** Wenping Song, Leike Li, Xuejia Liu, Yanhe Zhu, Shimin Yu, Haocheng Wang, Lin Wang

**Affiliations:** ^1^ State Key Laboratory of Robotics and System, Harbin Institute of Technology, Harbin, China; ^2^ Chongqing Research Institute of HIT, Chongqing, China; ^3^ Department of Medical Imaging, The Fourth Affiliated Hospital of Harbin Medical University, Harbin, China; ^4^ College of Engineering, Ocean University of China, Qingdao, China

**Keywords:** hydrogel, microrobots, preparation method, actuation mechanisms, biomedical application

## Abstract

Recent years have witnessed a surge in the application of microrobots within the medical sector, with hydrogel microrobots standing out due to their distinctive advantages. These microrobots, characterized by their exceptional biocompatibility, adjustable physico-mechanical attributes, and acute sensitivity to biological environments, have emerged as pivotal tools in advancing medical applications such as targeted drug delivery, wound healing enhancement, bio-imaging, and precise surgical interventions. The capability of hydrogel microrobots to navigate and perform tasks within complex biological systems significantly enhances the precision, efficiency, and safety of therapeutic procedures. Firstly, this paper delves into the material classification and properties of hydrogel microrobots and compares the advantages of different hydrogel materials. Furthermore, it offers a comprehensive review of the principal categories and recent innovations in the synthesis, actuation mechanisms, and biomedical application of hydrogel-based microrobots. Finally, the manuscript identifies prevailing obstacles and future directions in hydrogel microrobot research, aiming to furnish insights that could propel advancements in this field.

## 1 Introduction

In recent years, the burgeoning advancements in microrobots have marked a significant milestone in the trajectory of future precision medicine (Li et al., 2017; Xu et al., 2017). Characterized by their diminutive size and adaptable morphology, microrobots can navigate complex and confined spaces, executing tasks beyond the reach of conventional macro-scale robots (Chen et al., 2022). Such innovations herald unprecedented opportunities for precision medicine (Li et al., 2023), encompassing precise drug delivery (Medina-Sánchez et al., 2018; Srivastava et al., 2019; Wang et al., 2023), tissue repair (Fedorovich et al., 2008), and disease diagnosis (Wen et al., 2014; Shin et al., 2015), among others. Nonetheless, the path to the widespread integration of microrobots into clinical settings is fraught with challenges, notably concerning the degradability and biosafety of the materials employed (Noh et al., 2022; Zhao et al., 2022). Within this framework, hydrogel-based microrobots emerge as a formidable solution, leveraging their exceptional biocompatibility, customizable physico-mechanical attributes, and acute sensitivity to biological environments. This positions hydrogel microrobots as a pivotal enabler, charting novel avenues for the deployment of microrobots within healthcare domains.

Within the biomedical field, hydrogel microrobots have progressively evolved, advancing from foundational motor capabilities to an adeptness at navigating complex biological conditions and executing precise medical interventions. Initial developments in hydrogel microrobots centered on endowing them with fundamental motility, utilizing external stimuli like magnetic fields, light, or ultrasound for propulsion. A notable example includes the drug-encapsulating poly (lactic-co-glycolic acid) (PLGA) magnetic microspheres (Gao et al., 2012), engineered for both rotational and linear movements under the modulation of an external magnetic field. This foundational functionality sets the stage for the execution of more intricate tasks.

As technological progress forged ahead, the focal point of research pivoted to the hydrogel microrobots’ capacity to intelligently respond to specific shifts within biological settings—ranging from pH fluctuations and thermal variations to the identification of unique biomolecules. Such advancements permit the microrobots to autonomously adjust their actions in response to the biological milieu, facilitating, for example, the triggered release of pharmaceuticals when encountering designated biological signals (Maria-Hormigos et al., 2023). Particularly, a bio-degradable hydrogel-based micro-swimmer has been demonstrated to initiate the swift expansion of its internal hydrogel network in reaction to MMP-2 enzyme concentration variations, thus effectuating the liberation of encapsulated drugs (Ceylan et al., 2019).

Moreover, the refinement of hydrogel microrobots has equipped them to perform tasks of greater complexity and precision within medical scenarios. Illustrative of this is a groundbreaking multifunctional medical microrobot system that not only accomplishes liver chemoembolization but also supports real-time and post-procedural imaging through X-ray and magnetic resonance imaging (MRI) modalities. This system amalgamates a microrobot with a magneto-actuation module, incorporating a hydrogel-enshrouded porous architecture and magnetic nanoparticles. Leveraging magnetic navigation, the microrobot precisely targets tumor-feeding vessels, facilitating the direct application of transcatheter hepatic chemotherapy within a living organism (Go et al., 2022).

However, despite the tremendous potential of hydrogel microrobots, several challenges and dilemmas persist regarding new materials and drive technologies (Wang et al., 2021a). Firstly, the commonly employed hydrogel materials exhibit limitations in certain aspects, including mechanical strength, stability, and biodegradability. Consequently, there is a pressing need to explore novel materials that can overcome these limitations effectively. Secondly, the existing drive technologies still require enhancements to enable precise maneuvering and efficient actuation. In response to these challenges, numerous researchers are actively investigating and developing new materials and actuation technologies to foster the continued advancement and application of hydrogel microrobots (Soto et al., 2020; Oral and Pumera, 2023).

**FIGURE 1:**
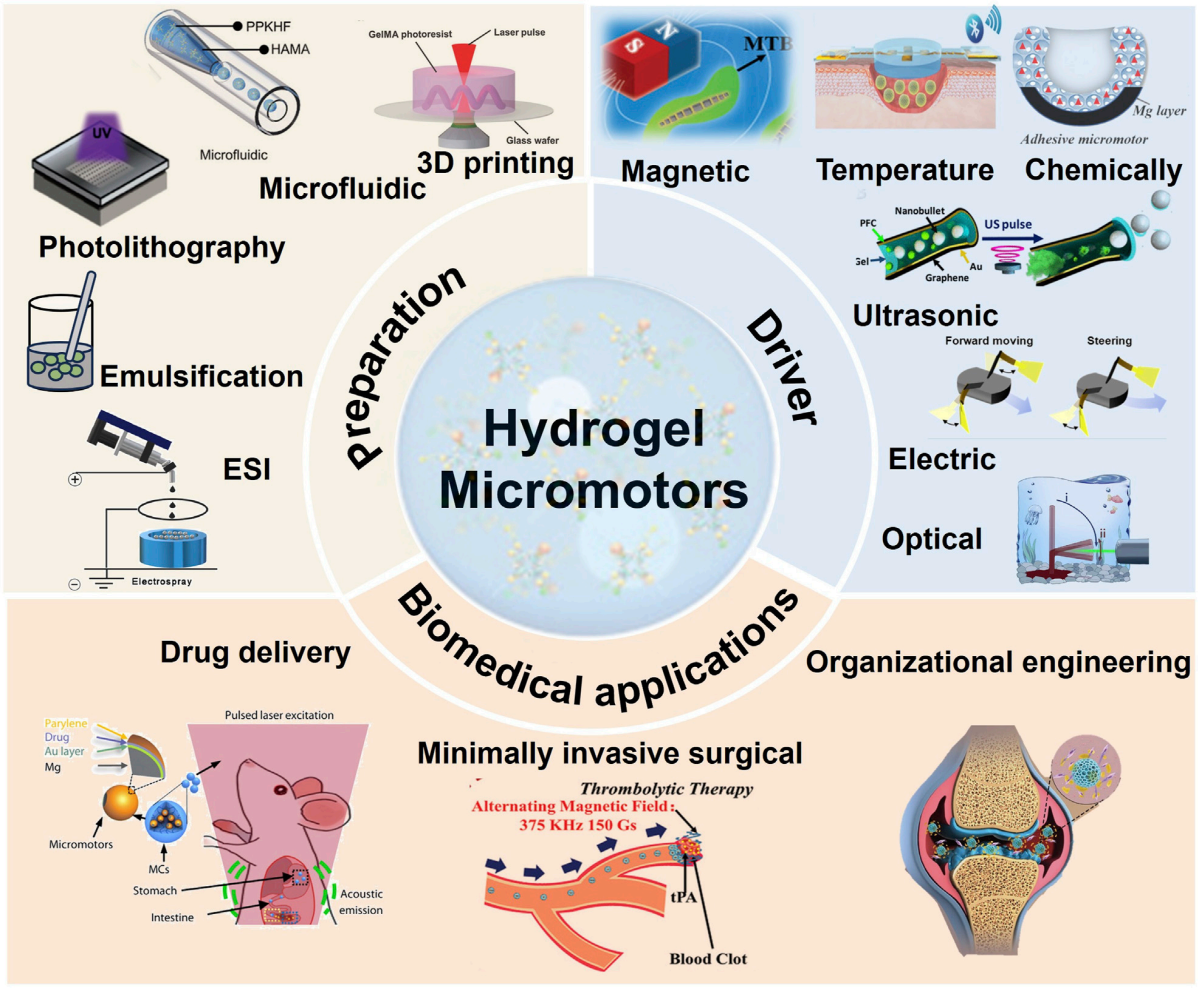
Preparation, driver and biomedical applications of hydrogel micromotors (Soto et al., 2016; Wang et al., 2018; Wu et al., 2019; Zhao et al., 2019; Zhong et al., 2019; Kim et al., 2020; Pang et al., 2020; Xie et al., 2020; Li et al., 2021; Cai et al., 2022; Ko et al., 2022; Yao et al., 2023; Bai et al., 2024). 3D printing, Copyright ^©^ 2018, John Wiley and Sons Microfluidic, Copyright ^©^ 2023 The Authors. Advanced Science published by Wiley-VCH GmbH Photolithography, Copyright ^©^ 2020, John Wiley and Sons ESI, Copyright ^©^ 2021 Acta Materialia Inc. Published by Elsevier Ltd. All rights reserved. Magnetic, Copyright ^©^ 2020, John Wiley and Sons Temperature, Copyright ^©^ 2020 The Authors. Published by WILEY-VCH Verlag GmbH and Co. KGaA, Weinheim Chemically, Copyright ^©^ 2021 The Authors. Advanced Science published by Wiley-VCH GmbH Ultrasonic, Copyright ^©^ 2016, American Chemical Society Electric, Copyright ^©^ 2022, The American Association for the Advancement of Science Optical, Copyright ^©^ 2019, The American Association for the Advancement of Science Minimally invasive surgical, Copyright ^©^ 2020, John Wiley and Sons Organizational engineering, Copyright ^©^ 2023, John Wiley and Sons.

This paper delivers an exhaustive review of the principal classifications and contemporary advancements in hydrogel microrobots, focusing on their fabrication techniques, actuation mechanisms, and application domains. Initially, the manuscript scrutinizes diverse classification approaches for multifunctional hydrogel materials. Subsequently, it offers an elaborate exposition of the fabrication methods, driving strategies for hydrogel microrobots, and their deployment in biomedical contexts. As shown in Figure 1, the article presents a concise summary, addressing the prevailing challenges and prospective pathways for advancements in hydrogel microrobots.

## 2 Types of multifunctional hydrogel materials

### 2.1 Classification based on source

(1) Natural Hydrogels: Originating from natural sources, these hydrogels are distinguished by their exceptional water absorption capabilities and gel-like properties, crafted from natural substances. Typically, these hydrogels consist of natural polymeric compounds renowned for their excellent biocompatibility and biodegradability. Notable examples encompass polysaccharides such as agarose, hyaluronic acid, alginate, and chitosan (Chelu et al., 2022), alongside proteins including fibronectin, sericin, collagen, and gelatin (Mano et al., 2007; Zhao et al., 2013).
(2) Synthetic Hydrogels: Fabricated from synthetic polymers, these hydrogels offer the advantage of precise control over chemical composition and the versatility of mechanical properties (Nasution et al., 2022). Examples include polyvinyl alcohol (PVA) (Chong et al., 2013), polyethylene glycol (PEG) (Lin and Anseth, 2009), and poly (*N*-vinyl caprolactam) (PNVCL) (Ramos et al., 2012). The physicochemical and functional attributes of these synthetic hydrogel materials can be finely adjusted by varying the synthesis techniques and material compositions, allowing for a broad range of applications tailored to specific requirements.

### 2.2 Classification based on preparation

(1) Physical Cross-linking: Hydrogels that undergo physical cross-linking evolve into gel structures through diverse mechanisms, including physical (hydrogen) bonding, crystallization, ionic interactions, self-assembly of small molecules, and mechanical dispersion (Chelu and Musuc, 2023). Notable examples include hyaluronic acid (Ye et al., 2017), poly (Wang et al., 2015), chitosan (Ishii-Mizuno et al., 2017), etc. These hydrogels are characterized by their ease of availability, obviation of cross-linking agents, reversibility, biocompatibility, and adjustable properties, positioning them as advantageous for various applications.
(2) Chemical Cross-linking: Gels synthesized through chemical cross-linking result from the covalent bonding of polymer chains, engendering a permanent network structure, hence their designation as irreversible gels (Caló and Khutoryanskiy, 2015). Notable examples include whey protein (Abaee et al., 2017), kolliphor (Varaprasad and Sadiku, 2015), cellulose nanofiber (Kobe et al., 2016), etc. The preparation of these chemically cross-linked gels typically involves a range of reactions, including photopolymerization (Nezhad-Mokhtari et al., 2019), enzymatic processes (Sperinde and Griffith, 1997), and click chemistry (Liu et al., 2022), ensuring a robust and enduring gel framework.

### 2.3 Types of stimulus-responsive hydrogels

(1) PH-responsive Hydrogels: PH-responsive hydrogels represent a class of adaptive and controllable materials, whose structural configuration and physical attributes can be finely tuned in response to the environmental pH (Gupta et al., 2002). Illustratively, the integration of pH-responsive hydrogels with 4D laser printing technologies has facilitated the development of magnetic microrobots capable of targeted drug delivery at precise locations (Xin et al., 2021). Furthermore, this innovative material has been applied in the creation of versatile microneedle sensing patches, which allow for the visual monitoring of wound pH levels (Xiao et al., 2024). Additionally, pH-responsive hydrogels have found extensive applications across a variety of medical devices, including soft microrobots (Li H. et al., 2016) and intelligent capsule switches (Han et al., 2020), demonstrating their broad potential in advancing healthcare.
(2) Temperature-responsive Hydrogel: Temperature-sensitive hydrogels are materials that exhibit responsiveness to temperature fluctuations, characterized by their reversible swelling and shrinking capabilities at varied thermal conditions. For instance, chitosan, upon dissolution in water, transitions from a fluid polymer solution to a gelatinous state at approximately 32°C (Fu et al., 2018). PNIPAM-based brushes, for example, can respond reversibly to temperature changes (Tu et al., 2017). This unique feature of hydrogels allows for the modulation of drug release rates by adjusting the temperature.
(3) Light-responsive Hydrogels: Light-responsive hydrogels are materials engineered to undergo gelation or dissolution in response to alterations in light intensity, wavelength, or direction (Li et al., 2019). An illustrative example includes the application of a modified fumaramide, which facilitates the photo-switching of the material from a liquid state to a gel (Matsumoto et al., 2008). The utilization of light as a trigger for gelation endows these systems with the capability for spatial and temporal regulation of the process, thereby enhancing the precision and controllability of their functional responses.
(4) Electro-responsive Hydrogels: Electro-responsive hydrogels are smart materials that can regulate their gelation and dissolution processes through the manipulation of electric field intensity and orientation. These hydrogels are capable of dissolving, contracting, or bending when subjected to an electric field, effectively converting electrical energy into mechanical energy (Lin et al., 2021). This functionality endows the hydrogels with versatile applications, including sensor technology, controlled drug delivery, and the development of smart materials, thereby highlighting their significant potential in various technological domains (Mirvakili and Langer, 2021).
(5) Chemical-responsive Hydrogels: Chemically responsive hydrogels are a class of gel materials that exhibit sensitivity to the presence or variation in concentration of particular chemicals, undergoing reactions that result in gelation or dissolution. Notably, these hydrogels can be engineered to incorporate glucose-responsive elements into systems for insulin or insulin delivery, facilitating bioactive release (Fuchs et al., 2021). This property underscores their potential in creating precise, chemically triggered delivery mechanisms, particularly in biomedical applications such as controlled drug release.

## 3 Preparation method of hydrogel microrobots

### 3.1 Microfluidic methods

Microfluidics involves the manipulation of immiscible fluids within microscale channels on microfluidic chips, leading to the formation of droplets at channel intersections (Daly et al., 2020). These droplets are subsequently cross-linked to produce hydrogel microspheres. The application of microfluidic flow techniques for the synthesis of hydrogel microspheres facilitates enhanced precision, enabling the achievement of a homogeneous particle size distribution. In 2022, Shao et al. (Shao et al., 2022) introduced an accessible microfluidic device, constructed by integrating a commercially available needle with a sharp tapered end into transparent silicone tubing. This innovation is tailored for the fabrication of hydrogel microspheres, characterized by their uniform and adjustable dimensions. The device’s design is notably user-friendly, simplifying its operation and enhancing control over the process. By adjusting the flow of the liquid and the sharpness of the needle, it is possible to reliably produce hydrogel microspheres with consistent sizes and customizable dimensions.

### 3.2 Emulsification method

Emulsification entails the blending of immiscible liquids, such as water and oil, to produce cross-linkable hydrogel droplets through agitation. A representative application of this technique is the fabrication of hydrogel microspheres via the formation of an interpenetrating polymer network between chitosan and polyacrylamide-grafted guar gels (Kajjari et al., 2011). This process underscores the versatility of emulsification in enabling the synthesis of complex hydrogel structures, highlighting its significance in the development of advanced materials with tailored properties.

### 3.3 3D printing

To fabricate miniature hydrogel robots featuring intricately machined structures and sophisticated geometries, researchers have employed 3D printing technologies (Wallin et al., 2018). Specifically, two-photon polymerization (TPP)-based 3D printing has been utilized for the nanoscale precision manufacturing of three-dimensional helical structures, endowing gelatin methacrylate (GelMA) hydrogel robots with precisely defined properties and functionalities (Wang et al., 2018). This advanced technology affords a highly precise and controllable approach to the construction of hydrogel robots, heralding a new era in the development of customized functional robotics.

### 3.4 Photolithography

Photolithography, a sophisticated technique, employs photomasks with micron- or nanometer-scale resolution to engineer intricate structures (Tasoglu et al., 2014). Materials such as polydimethylsiloxane (PDMS), polyurethane (PU), and polyimide are exemplary in serving as templates for the construction of robotic body structures (Kim et al., 2020). These templates are pivotal in bestowing the resulting robotic frameworks with flexibility, customizability, and durability, thereby enhancing the functional and structural integrity of the robots.

### 3.5 Electrospray ionization

Electrospray ionization (ESI) is a technique driven by potentiodynamic forces for the atomization of liquids, facilitating the synthesis of hydrogel microspheres through electrostatic forces and ionic cross-linking agents (Chi et al., 2023). This method offers an efficient approach to tailoring particle size by fine-tuning experimental parameters, enabling the production of uniform hydrogel microspheres with diameters ranging from several micrometers to thousands of micrometers. During the process, a gel solution is atomized into minuscule droplets under the application of high voltage, which are then ionized instantaneously. The material within these droplets undergoes cross-linking or gelation reactions, culminating in the formation of solid hydrogel microspheres (Li et al., 2021). By manipulating the settings of the ESI system, it is possible to precisely adjust the characteristics of the microspheres, thus accommodating a diverse array of application requirements.

Table 1 in this study summarizes the advantages and disadvantages of various methods for preparing hydrogel microrobots. Key metrics for comparison include preparation efficiency, dimensional accuracy, size adjustability, and material suitability. Among the methods, microfluidic and electrospray ionization exhibit high efficiency and broad applicability. 3D printing and photolithography offer excellent dimensional accuracy, although photolithography has limitations in terms of dimensional tunability. The emulsification method is relatively simple but lacks precision and material tunability. Selection of the appropriate preparation method should be based on specific application requirements and practical considerations.

**TABLE 1:** Comparison table of hydrogel micromotor preparation methods.

Preparation method	Efficiency	Dimensional accuracy	Size adjustability	Material suitability
Microfluidic	★★★★	★★★	★★★★	★★★★
Emulsification	★★★	★	★★	★★
3D printing	★★	★★★★	★★★★	★★
Photolithography	★★	★★★★	★	★★
ESI	★★★★	★★	★★	★★★★

## 4 Hydrogel microrobot driver

### 4.1 Magnetic drive

The prospect of magnetic propulsion in microrobots (Li et al., 2018; Yu et al., 2022; Zhang et al., 2023), highlighted by its capacity for small-scale remote control, holds considerable promise across various domains (Ji et al., 2021; Yu et al., 2024). Utilizing magnetic field actuation (Wang et al., 2024b), an array of applications has been explored, including the deployment of hydrogel-based microrobot end-effectors. These end-effectors are adept at executing precision tasks such as targeting, release, and sampling, governed by magnetic fields in conjunction with ambient ionic stimuli (Zheng et al., 2021). The precision afforded by magnetic actuation, coupled with its minimal impact on biological tissues, has led to the pioneering strategy that achieves, for the first time, the fully independent and decoupled control of up to four flexible thin-film magnetic microrobots within a single, uniformly oscillating magnetic field (Xu et al., 2022). Moreover, the exploration of magnetically driven micro-populations has emerged as a focal research area. For instance, the application of laser scatter-contrast imaging enables the real-time tracking of magnetic nanomachine populations within blood vessels, facilitating their guided navigation through the intravascular space (Wang et al., 2024a), thus opening new frontiers in medical and robotic research.

### 4.2 Optical drive

Light is currently one of the most common tools for wireless power supply and control of mobile microscale devices, so light is widely used to drive microrobots (Yang W. et al., 2023). Magnetic and optical actuation methods are the most widely used actuation methods in mobile microrobots currently (Sitti and Wiersma, 2020). Leveraging the capabilities of light-responsive hydrogels, as previously discussed, these microrobots are capable of executing reversible and repeatable motions with instantaneous response to light, all under the convenience of wireless remote control (Hou et al., 2023). Among these, photocatalyst-based photocatalytic microrobots stand out as a pivotal category of light-driven microrobots (Dong et al., 2018). These motors harness energy from both an external light source and the surrounding chemical environment to achieve efficient propulsion. Furthermore, light-responsive actuators have emerged (Zhao et al., 2019), utilizing variations in light intensity to facilitate precise control over motion and morphological adjustments. The rapid response and versatile adaptability of light-responsive hydrogels pave the way for innovative applications, ranging from motion control in microrobots to the dynamic shape modulation of wearable technologies, offering groundbreaking solutions in these domains.

### 4.3 Electric field drive

The electric field actuation method operates without the need for additional fuel sources, primarily employing DC or AC electric fields for propulsion (Wang et al., 2021b). Researchers have innovatively incorporated a pleated nanomembrane electrode onto the external surface of the hydrogel robot (Ko et al., 2022). This electrode is crafted from metal nanoparticles, assembled through capillary forces, and induced by solvation. The resulting pleated configuration demonstrates superior mechanical deformation capabilities along with heightened electrical conductivity, enhancing the robot’s performance and responsiveness to electric fields.

### 4.4 Chemically drive

Chemical propulsion mechanisms in microrobots predominantly leverage the creation of localized concentration gradients, potential gradients, and the generation of bubbles via chemical reactions with specific chemical fuels to facilitate navigation through fluidic environments (Wang et al., 2013; Li J. et al., 2016; Moran and Posner, 2017). For instance, a hydrogel-based micromotor designed for stomach drug delivery exemplifies this principle (Cai et al., 2022). This micromotor, endowed with adhesive capabilities and loaded with magnesium (Mg), initiates spontaneous movement upon exposure to gastric juices. This activity is driven by the continuous production of hydrogen bubbles resulting from the Mg/H^+^ reaction. Such a micromotor efficiently binds to ulcerated regions, where it subsequently administers the therapeutic agent, thereby achieving the intended therapeutic effect. Despite these advancements, challenges related to the long-term stability of the micromotor and the precise modulation of drug release remain unresolved, indicating areas for future refinement and research.

### 4.5 Temperature drive

Drawing upon the principles of the previously discussed temperature-responsive hydrogel, a material sensitive to temperature can be integrated as a constituent of the supramolecular nanomotor. This allows the nanomotor to undergo morphological or property alterations within a defined temperature range, facilitating the modulation of its motion speed (Tu et al., 2017). Both supramolecular nanomotors and temperature-responsive materials represent dynamic fields of inquiry, harboring promising prospects for applications in precision-controlled cargo transportation (Pang et al., 2020).

### 4.6 Ultrasonic drive

Ultrasonic field actuation leverages ultrasound waves to precisely control and propel microrobots, offering a non-invasive, demand-driven motion capability with extended durability and excellent biocompatibility. Utilizing ultrasonic energy as an external stimulus, researchers have devised a miniature cannon robot incorporating a gelatin-based hydrogel matrix (Soto et al., 2016). This matrix is embedded with perfluorocarbon emulsion and nano-bullets, assembled through ultrasound activation. The technology facilitates deep tissue penetration by activating perfluorocarbon emulsions and nano-bullets, which are engineered to carry and precisely deploy multiple payloads. Such advancements herald the significant potential for applications in medical diagnostics, therapeutic delivery, and logistics, reflecting the versatile utility of ultrasonic actuation in advancing microrobot technologies.

Table 2 provides a comprehensive overview of the advantages and disadvantages of the driving methods mentioned above. Key metrics for comparing hydrogel microrobot actuation methods include exposure, response, efficiency, and actuation device volume. All the described methods are contactless. Magnetic drive, electric field drive, and ultrasonic drive exhibit faster response speeds and higher actuation efficiency. Chemically drive and temperature drive methods are less efficient. Regarding actuation device volume, chemical drives do not require specialized drive units and primarily rely on chemical substances for actuation, while magnetic drives necessitate larger magnetic field devices and, consequently, larger drive units. Choosing the appropriate drive method is crucial based on specific application requirements and practical considerations.

**TABLE 2:** Comparison table of hydrogel micromotor drive methods.

Drive method	Exposure	Response	Efficiency	Driver volume
Magnetic drive	×	★★★★	★★★★	★★★★
Optical drive	×	★★★	★★	★★
Electric field drive	×	★★★★	★★★	★★★
Chemically drive	×	★★★	★	×
Temperature drive	×	★★	★	★★
Ultrasonic drive	×	★★★★	★★★★	★★★

## 5 Biomedical applications of hydrogel microrobots

### 5.1 Drug delivery

Hydrogel particles, notably microspheres, are extensively utilized in drug delivery systems due to their exceptional modifiability, enabling their application as controlled release mechanisms that enhance the bioactivity and stability of pharmaceuticals. Their versatility allows for their use across various modalities, including injectable formulations, targeted delivery systems, topical applications, and oral medications (Karshalev et al., 2018). Through the meticulous design and adjustment of hydrogel particle properties, it is feasible to attain a precisely controlled drug release, thereby augmenting therapeutic outcomes, minimizing adverse effects, and reducing medicinal wastage. This strategic approach underscores the potential of hydrogel-based technologies in advancing drug delivery science.

**FIGURE 2:**
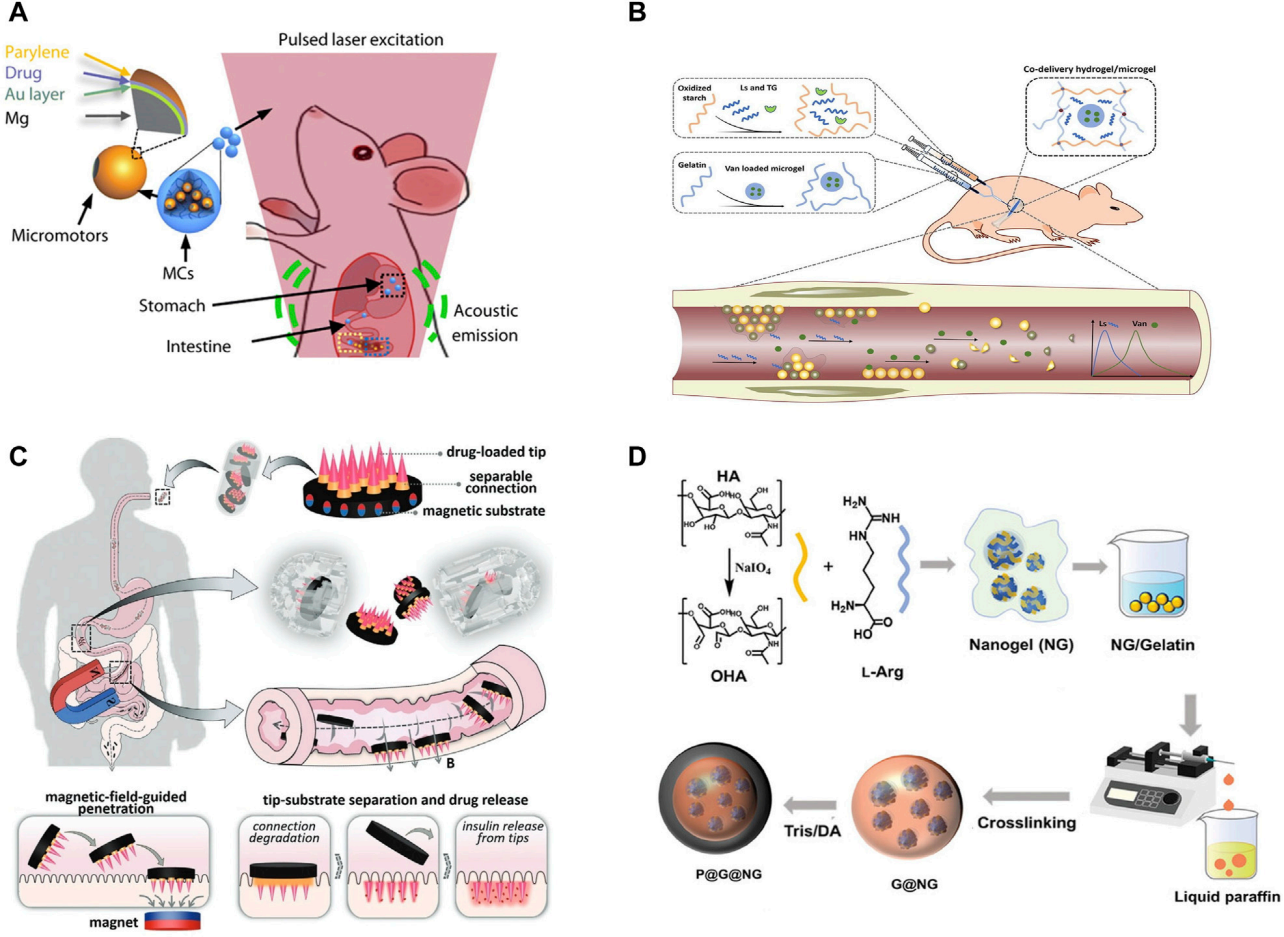
**(A)** Schematic of the PAMR in the GI tract. The MCs are administered into the mouse. NIR illumination facilitates the real-time PA imaging of the MCs and subsequently triggers the propulsion of the micromotors in targeted areas of the GI tract (Wu et al., 2019) Copyright ^©^ 2019, the American Association for the Advancement of Science. **(B)** An injectable hydrogel/microgel co-delivery system (Cheng et al., 2022). **(C)** Schematic illustrations of composition, release, drug delivery, and operational principle of the oral magneto-responsive microneedle robots (Zhang et al., 2021). **(D)** Preparation of P@G@NG microcapsules (Fu et al., 2024). **(A)** Copyright ^©^ 2019, The American Association for the Advancement of Science **(B)** Copyright ^©^ 2022 Elsevier **(B)** V. All rights reserved. **(C)** Copyright ^©^ 2023, John Wiley and Sons **(D)** Copyright ^©^ 2024, John Wiley and Sons.

Wang et al. introduced an advanced micromotor system depicted in Figure 2A, featuring a micromotor encapsulated within an enteric gelatin capsule (Wu et al., 2019). This capsule is adeptly navigated through the gut *in vivo*, under the guidance of photoacoustic computed tomography (PACT). This ingenious integration facilitates the micromotor’s resistance to stomach acid, thereby averting any premature chemical interactions. Upon the microrobot’s arrival at a predetermined gut location, it triggers a gel-to-sol phase transition within the gelatin-based capsule through sustained near-infrared irradiation, culminating in the micromotor’s release. This pioneering integration of the microrobot system with PACT heralds a new era of deep-tissue imaging and meticulous control over *in vivo* microrobots. Significantly, this system not only enhances the retention time of pharmaceuticals within the organism but also supports the targeted and controlled release of medication.

Shi et al. developed microgels incorporating vancomycin (Van) utilizing a microfluidic emulsion technique, subsequently co-encapsulating these with lysostaphin (Ls) within a hydrogel matrix (Cheng et al., 2022). This resulted in an injectable hydrogel/microgel co-delivery system, depicted in Figure 2B, endowed with both bactericidal and biofilm-dispersing functionalities. The synergistic action of the encapsulated agents enhances bactericidal efficacy, while the injectable format of the hydrogel system facilitates ease of application for topical treatments, ensuring precise drug delivery to targeted areas. This research offers innovative perspectives and methodologies for advancing antimicrobial therapies.

Zhao et al. engineered a magnetically responsive microneedle robot designed for the efficient oral administration of multifunctional macromolecules (Zhang et al., 2021). This innovative robotic system comprises a magnetic base, detachable links, and insulin-imbued tips, as depicted in Figure 2C. The magnetic foundation utilizes poly (ethylene glycol) diacrylate (PEGDA) infused with magnetizable NdFeB particles. The detachable links are crafted from BSA and a minimal concentration of GelMA, whereas the tips are formed from GelMA-containing insulin. These microneedle robots are then encapsulated within standard enteric capsules. Strategic magnetization of the magnetic base allows for precise guidance of the microneedle tip to the small intestine wall. Upon engagement with the tissue, the detachable links are designed to slowly disintegrate, facilitating the targeted release of insulin. This mechanism, through magnetic guidance coupled with the biodegradation of the separable links, affords meticulous insulin delivery, presenting a promising avenue for diabetes management.

Yang et al. introduced a novel gas therapy approach for treating Inflammatory Bowel Disease (IBD), focusing on mitigating inflammation through the modulation of intestinal microbiota (Fu et al., 2024). This approach centers on the creation of a nanogel-based, multi-stage nitric oxide (NO) delivery system designed to target inflamed tissues precisely, enabling site-specific NO release. The process initiates with the reaction between the aldehyde group of modified oxidized hyaluronic acid (OHA) and the dual amine groups of L-Arginine (L-Arg), forming nanogels (NGs) through Schiff base reaction assembly. These NGs are then microencapsulated within gelatin microspheres and surface-modified to produce gelatin@nanogel (G@NG) microcapsules. A further layer of polydopamine (PDA) is coated around these to form PDA@Gel@Nanogel (P@G@NG) composite microcapsules, depicted in Figure 2D. The oral administration of these composite microcapsules facilitates the *in situ* and reactive release of NO directly at the inflammation sites within the colon, effectively modulating the gut microbiota and alleviating colitis symptoms. This research not only offers a targeted and efficient new treatment modality for IBD but also exemplifies the significant potential of nanotechnology in medical applications.

Hydrogel microrobots exhibit substantial potential in the realm of drug delivery, offering a dual advantage: protection and controlled release. Encased within a hydrogel shell, drugs are safeguarded from the hostile bodily environment, while the material’s inherent properties facilitate a modulated release. This ensures the sustenance of an efficacious drug concentration within the target therapeutic zone. Moreover, the hydrogel’s responsive nature to environmental stimuli such as pH, temperature, or enzymatic activity allows for precision-targeted drug release. As carriers, hydrogel microrobots not only present numerous benefits but also pose significant challenges, including the accuracy and reliability of their control mechanisms, alongside the imperative to surmount obstacles related to large-scale fabrication and the complexities of clinical adoption.

### 5.2 Organizational engineering

Hydrogel microrobots have garnered widespread recognition in tissue engineering and regenerative medicine, attributed to their outstanding biocompatibility and biodegradability. The triad of tissue engineering—comprising seed cells, scaffolds, and growth factors—plays a pivotal role in facilitating tissue regeneration and repair. Hydrogel microrobots align seamlessly with these three fundamental components, demonstrating promising potential and beginning to establish a significant presence in the domain of tissue repair.

**FIGURE 3:**
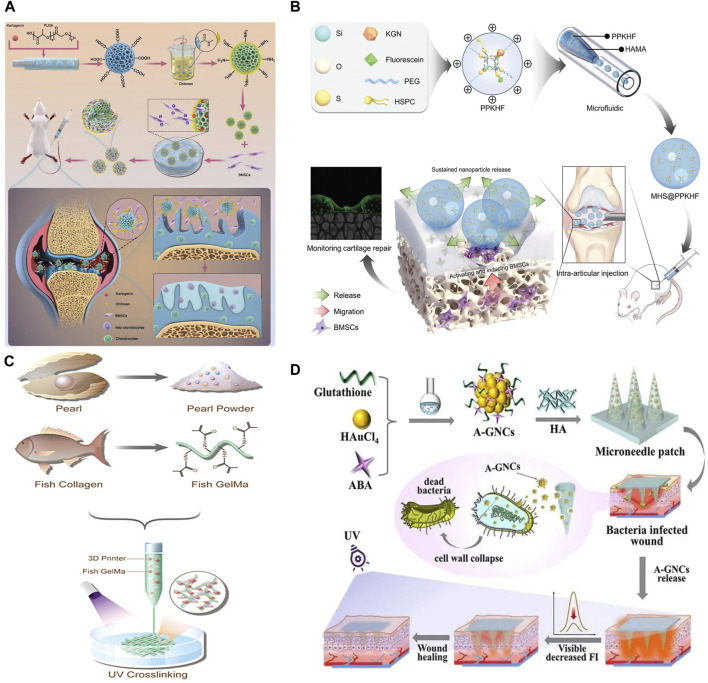
**(A)** Schematic illustration of Preparation and application of BMSCs Delivery system (Bai et al., 2024). **(B)** A micro-nano double lubricating hydrogel microsphere with functions of *in situ* cartilage regeneration and repair process visualization (Yao et al., 2023). **(C)** The composition and microfluidic 3D printing of PP hybrid bioactive scaffold (Yang L. et al., 2023). **(D)** Schematic illustration of the aminobenzeneboronic acid-modified gold nanoclusters (A-GNCs) encapsulated fluorescent microneedles array applied to the bacteria-infected skin wounds (Yi et al., 2024). **(A)** Copyright ^©^ 2023, John Wiley and Sons **(B)** Copyright ^©^ 2023 The Authors. Advanced Science published by Wiley-VCH GmbH **(C)** Copyright ^©^ 2023 The Authors. Advanced Science published by Wiley-VCH GmbH **(D)** Copyright ^©^ 2024 The Authors. Aggregate published by South China University of Technology; AIE Institute and John Wiley and Sons Australia, Ltd.

Lin et al. encapsulated chitosan (CS) within porous PLGA microspheres (Bai et al., 2024), culminating in the development of PLGA-CS microspheres as illustrated in Figure 3A. These microspheres are characterized by their exceptional mechanical robustness, which serves to shield mesenchymal stem cells (MSCs) from shear-induced trauma. Additionally, they facilitate the differentiation of MSCs into chondrocytes and stimulate the expression of cartilage repair genes via the integration of kartogenin (KGN). The study reveals that PLGA-CS@KGN microspheres markedly enhance the differentiation process of MSCs into chondrocytes, diminish osteoid volume, and mitigate osteoarthritis (OA) progression in rats. PLGA-CS@KGN microspheres possess high loading capacity and injectability, indicating potential for tissue regeneration. They represent a novel, minimally invasive therapeutic strategy for OA treatment, underscoring the pivotal role of stem cell tissue engineering in addressing osteoarthritis.

Adopting the principle of integrated treatment monitoring, Yao et al. developed micronized, double-lubricated hydrogel microspheres designed to enable the visualization of *in situ* cartilage regeneration and repair processes, thus facilitating the monitoring of tissue regeneration (Yao et al., 2023), as depicted in Figure 3B. This study introduced methacrylate hyaluronate microspheres (MHS@PPKHF) that undergo gradual degradation and enable the controlled release of PPKHF, synthesized via microfluidics for managing and monitoring osteoarthritis (OA). These microspheres can be administered directly into the joint cavity. Incorporating fluorescent imaging capabilities, the microspheres not only support cartilage repair but also improve joint lubrication. This innovative approach represents a significant advancement in the field of regenerative medicine, offering a promising tool for the effective treatment and real-time monitoring of osteoarthritis.

Yu et al. selected pearl powder (PP) (Yang L. et al., 2023), a bioactive material with a composition analogous to bone tissue, and amalgamated it with fish skin GelMA and vascular endothelial growth factor (VEGF) to fabricate a composite scaffold. This scaffold is engineered to facilitate bone regeneration, as depicted in Figure 3C. Its application extends to clinical scenarios such as bone defect repair, fracture healing, and bone reconstruction, offering patients enhanced and sustainable treatment modalities. Nonetheless, the comprehensive assessment of the biocompatibility, bioactivity, and therapeutic effectiveness of these composite scaffolds necessitates further investigation and clinical validation.

Wound healing remains a critical issue in clinical and healthcare environments. Zhao et al. innovated an encapsulated nanomedicine microneedle array designed for the intelligent management of wounds infected by bacteria (Yi et al., 2024). This advancement involved the preparation of hyaluronic acid (HA) microneedle patches, achieved by encapsulating aminophenylboronic acid-modified gold nanoclusters (A-GNCs) specifically for the treatment of infected wounds, and loading these onto PDMS negative mold replicated HA microneedle substrates, as illustrated in Figure 3D. The A-GNCs microneedle patches are engineered to breach wound tissues, while the subsequent degradation of HA facilitates the targeted release of A-GNCs, thereby exerting antibacterial effects. Furthermore, this research demonstrates that the microneedle patches exhibit three essential functions in the treatment of *Staphylococcus* aureus-infected wounds: bacterial suppression, autonomous monitoring of residual drug levels, and enhancement of wound healing processes.

Hydrogel microrobots have emerged as a transformative tool in the field of tissue engineering, showcasing their profound ability to facilitate tissue repair and regeneration. Hydrogel microrobots exhibit remarkable biocompatibility, enabling seamless integration with seed cells and mitigating potential immune responses. Moreover, these microrobots serve as scaffolds, providing a conducive environment for cellular growth. Additionally, they can effectively transport growth factors, facilitating the targeted release of drugs or growth factors. This capability plays a crucial role in promoting cell proliferation, facilitating cellular differentiation, and aiding in tissue repair processes (Jiang et al., 2022). Consequently, hydrogel microrobots have introduced innovative strategies for tissue repair and regeneration. Future endeavors in this domain should concentrate on augmenting the performance and usability of these microrobots within biological systems. Additionally, overcoming hurdles related to clinical translation remains imperative to unlock the full potential of hydrogel microrobots in tissue engineering applications.

### 5.3 Minimally invasive surgical

Miniature hydrogel-based robots represent a cutting-edge technological advancement, capable of being actuated by external stimuli to accurately navigate to specific locations to conduct minimally invasive medical procedures or treat diseases.

**FIGURE 4:**
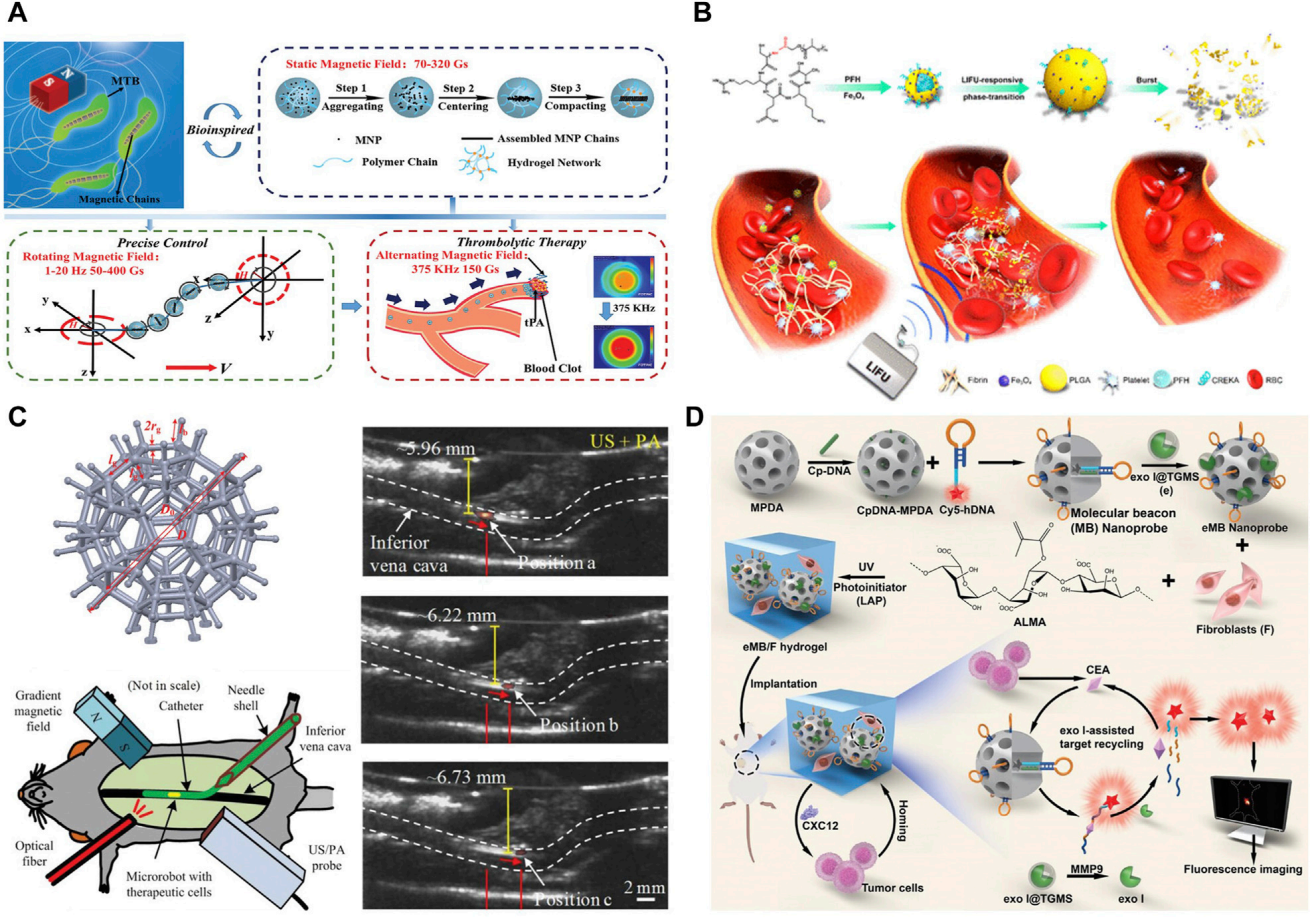
**(A)** Schematic representation of the BMM with magneto-collective regulation for targeted thrombolysis (Xie et al., 2020). **(B)** The mechanism by which Fe3O4-PLGA-PFH-CREKA NPs nanoparticles facilitate the dissolution of blood clots (Zhong et al., 2019). **(C)** Calculation of the model of the burr-like porous microrobot with optimized size and Image-guided navigation of cell-loaded microrobots in the inferior vena cava of the mouse and its position error (Wei et al., 2020). **(D)** The preparation of the eMB/F hydrogel sensor and its *in vivo* ultrasensitive optical imaging mechanism of target CEA of residual microtumors (Geng et al., 2024). **(A)** Copyright ^©^ 2020, John Wiley and Sons **(B)** Reprinted (adapted) with permission from (Low-Intensity Focused Ultrasound-Responsive Phase-Transitional Nanoparticles for Thrombolysis without Vascular Damage: **(A)** Synergistic Nonpharmaceutical Strategy). Copyright (2019) American Chemical Society. **(C)** Copyright ^©^ 2020, John Wiley and Sons. **(D)** Copyright ^©^ 2024, John Wiley and Sons.

Microrobots, renowned for their precise targeting capabilities, are being explored for their potential to disintegrate thrombi at various locations through the deployment of micro- and nanoscale swarms. Among the forefront of current research endeavors is thrombolytic therapy facilitated by magnetic hydrogel robots. Wang et al. engineered a cohort of diminutive hydrogel-based robots for disease treatment (Xie et al., 2020), as illustrated in Figure 4A. These robots are imbued with magnetic properties and are equipped to transport tissue plasminogen activator (tPA). Propelled by a rotating magnetic field, these magnetic spherical robots are capable of navigating to a thrombus within an artificial vascular model. Upon congregating at the thrombotic site, the cluster of robots initiates magnetic hyperthermia through alternating magnetic fields, concurrently releasing tPA to effectuate thrombolysis.

Ran et al. developed a multifunctional type of nanoparticles (NPs), specifically Fe_3_O_4_-PLGA-PFH-CREKA NPs (Zhong et al., 2019), leveraging the highly biocompatible polymer PLGA as a carrier. Figure 4B conceptually depicts the mechanism by which these targeted thrombolytic nanoparticles facilitate the dissolution of blood clots. Moreover, incorporating iron oxide enables these NPs to serve dual functions, allowing for both magnetic resonance (MR) and photoacoustic (PA) imaging. This study not only pioneers targeted thrombolytic therapy for the treatment of blood clots but also integrates follow-up monitoring capabilities. By marrying the nanoparticles with multimodal imaging techniques, this approach promises a more precise and tailored strategy for thrombosis management.

Accurate localization, surveillance, and feedback mechanisms in small hydrogel-based robots are essential for achieving targeted therapeutic interventions with closed-loop control (Chen et al., 2021). Advances in hydrogel-based microrobots have extended to imaging-guided surgery, exemplified by the development of a PEGDA-based hydrogel microrobot (Wei et al., 2020). This robot features a burr-like porous spherical architecture, optimized for real-time *in vivo* imaging, as depicted in Figure 4C. Leveraging photoacoustic imaging, these microrobots, embedded with cells, can be visualized and precisely directed towards a specified site under a gradient magnetic field. Moreover, these microrobots are designed to autonomously release their cellular payload, which can actively impede tumor proliferation, showcasing a novel approach to cell-based therapy.

*In vivo* optical imaging for the detection of trace biomarkers within residual microscopic tumors is crucial for enhancing cancer prognosis, yet it presents a significant challenge. Liu et al. innovated a novel hydrogel sensor (Geng et al., 2024), as illustrated in Figure 4D. This sensor is tailored for the ultra-sensitive and specific imaging of elusive biomarkers, heralding advancements in patient outcomes following surgical cancer interventions.

Hydrogel microrobots have emerged as pivotal tools in minimally invasive surgery, offering capabilities for accurate positioning and manipulation. These properties enable their use in specialized medical applications, including targeted drug delivery and the detection of biomarkers. Additionally, the inherent biocompatibility and biodegradability of hydrogel materials assure the *in vivo* safety of these microrobots. Nonetheless, enhancing the operational stability and reliability of hydrogel microrobots remains a significant challenge, particularly in the precise control of their movement within complex biological environments. The continued development and refinement of hydrogel microrobots are anticipated to significantly augment surgical precision and diminish patient recovery periods, heralding innovative advancements in minimally invasive surgical practices.

## 6 Conclusion

This manuscript comprehensively explores the domain of hydrogel microrobots, beginning with a detailed categorization of hydrogels alongside an examination of their distinct properties. The diverse classes of hydrogels find application across various scenarios, attributed to their unique characteristics. Notably, hydrogels are celebrated for their tunability, controllability, and biocompatibility, making them an esteemed material choice. The discussion then extends to the fabrication and actuation strategies of hydrogel microrobots, revealing a spectrum of methodologies and actuation techniques that enhance the versatility and applicability of these microrobots in real-world settings. The manuscript culminates with an in-depth analysis of hydrogel microrobots within the biomedical sphere. Hydrogel microrobots emerge as pivotal tools in biomedical research, offering innovative solutions for drug delivery, targeted therapy, and tissue engineering. These applications underscore the transformative potential of hydrogel microrobots in advancing medical technologies and improving therapeutic interventions. Overall, this paper furnishes a comprehensive review of hydrogel microrobots, encapsulating the classification, properties, preparation, actuation, and biomedical applications of hydrogels, thereby laying a foundational theoretical and practical groundwork for the future development and deployment of hydrogel microrobots.

Hydrogel microrobots possess extensive applicational prospects that transcend beyond the biomedical arena. Within the realm of environmental monitoring, these microrobots stand out for their capability to detect and track pollutants in aquatic and terrestrial environments, offering real-time insights into environmental health and delivering swift, accurate data. Their ability to autonomously navigate through water or soil, powered by specific actuation mechanisms, facilitates the targeted sampling and surveillance of diverse locales. Beyond environmental applications, hydrogel microrobots also show promise in industrial settings, finding utility in microfluidics, micromanipulation, and micromachining sectors for the execution of precise operations at microscales. As technological advancements persist and research progresses, the significance of hydrogel microrobots is set to amplify, heralding a new era of innovation and advancement across various disciplines.

Hydrogel microrobots, while harboring significant potential, encounter a range of challenges that must be addressed to unlock their full capabilities.

(1) Multifunctionality: Beyond singular-function devices, a key development goal for hydrogel microrobots is to attain multifunctionality. This includes the creation of hydrogel-based wearables that integrate multiple functions, such as biomonitoring, drug delivery, and therapeutic interventions within a single device. Multitasking capabilities, enabling simultaneous drug delivery, image acquisition, and lesion detection, necessitate the strategic design and integration of various functional modules to ensure cohesive operation and mutual enhancement.
(2) Processability: Although hydrogel microrobots can be relatively straightforward to fabricate, refining the control and repeatability of their preparation processes remains a challenge. This encompasses material selection, structural control, and morphological design. Enhancing preparation and processing methods is crucial for ensuring the consistency and stability of microrobots, making them suitable for diverse applications.
(3) Environmental Sustainability: Hydrogel microrobots are known for their biocompatibility and degradability, aligning with environmental sustainability principles. The design and preparation processes must prioritize the use of renewable, biodegradable materials and seek to minimize energy consumption and waste production, thereby ensuring the microrobots’ sustainability and reducing their ecological footprint.
(4) Programmability: The flexibility and adaptability of hydrogel microrobots call for consideration of their stability and precision upon activation. Programmability entails precise control over microrobot operations through external signals or controllers, requiring real-time feedback mechanisms that can dynamically adjust to various task demands, leveraging the stimulus-responsive nature of hydrogels.
(5) Stimulus Selectivity: Given the inherent stimulus-responsive characteristics of hydrogels, microrobots need to discern and selectively respond to specific stimuli—be it temperature, light, or electric fields. This selectivity enables precise movement, actuation, and targeted manipulation within specific environments.
(6) Driving Efficiency: The inherent softness and low density of hydrogel materials contribute to a reduction in energy conversion efficiency, resulting in lower propulsion efficiency. Moreover, the current methodologies for energy provision fail to supply adequate power, presenting obstacles in enhancing driving efficiency, ensuring sufficient power, and achieving precise control over the robotic movements.

In light of the aforementioned challenges, to advance hydrogel microrobots, it is necessary to undertake extensive research in the domains of new materials and drive technologies. The development of novel materials plays a pivotal role in achieving functional diversity and optimizing the performance of hydrogel microrobots. Researchers should focus on the exploration of new hydrogel materials with improved stability, controllability, and scalability for large-scale production. Additionally, enhancing drive technologies is crucial for augmenting the capabilities of hydrogel microrobots. While magnetron systems remain widely employed, they still present challenges in terms of precise maneuvering and speed control. Therefore, further research efforts and advancements in magnetron systems are imperative to achieve enhanced accuracy in positioning and control. By surmounting these hurdles, the field of hydrogel microrobots can propel forward, catalyzing innovation and expanding applications across medical, environmental, and industrial domains.
